# Treatment of Cerebellar Ataxia in the Context of Systemic Diseases

**DOI:** 10.1007/s11940-017-0485-y

**Published:** 2017-11-25

**Authors:** Malcolm Proudfoot, Alastair Wilkins

**Affiliations:** Institute of Clinical Neurosciences, University of Bristol, Southmead Hospital, Bristol, BS10 5NB UK

**Keywords:** Ataxia, Cerebellum, Systemic disease, Immunological disease, Paraneoplastic, Metabolic complications

## Abstract

*Purpose of review* The purpose of this review is to assess the evidence behind treatment regimens for cerebellar ataxias occurring in the context of systemic disease. We will address systemic conditions which are associated with specific involvement of the cerebellum (rather than widespread nervous system involvement) and those conditions for which some degree of evidence of treatment response exists.

*Recent findings* We have divided systemic disorders affecting the cerebellum into systemic immunological disorders, endocrine and metabolic disorders and paraneoplastic. Recent studies have increased understanding of the range of cerebellar disorders associated with a systemic immunological condition. The identification of newer pathogenic antibodies has improved diagnosis in conditions which would have previously been labelled as idiopathic. However, their rarity and phenotypic variability makes defining optimal immunomodulatory treatment regimens challenging. There is some evidence for beneficial effects of immunomodulation, particularly in anti-GAD ataxia and Hashimoto’s encephalopathy, although, at this time, specific treatment regimens cannot be defined. Immune-mediated paraneoplastic cerebellar disorders show response to therapy dependent, to some extent, on the underlying pathogenic antibody. Much is still to be understood concerning treatment regimens for the ataxic manifestations of metabolic disorders, notably alcohol-induced cerebellar injury, which are common and which are associated with significant disability.

*Summary* Despite their rarity, cerebellar ataxias occurring in the context of systemic disease cause significant morbidity and better therapies are required to improve outcomes associated with these conditions.

## Introduction

Dysfunction of the cerebellum can occur either as a result of localised injury or in the context of a number of systemic diseases. Systemic disorders often affect the central nervous system diffusely, but, in a number of situations, may specifically cause cerebellar dysfunction. Within this review, cerebellar ataxia in the context of systemic diseases will be considered. Broadly, systemic disorders affecting the cerebellum may be divided into systemic immunological disorders, endocrine and metabolic disorders and paraneoplastic. While some of the conditions discussed have no specific treatment, there are emerging studies suggesting specific conditions may respond to particular treatments.

## Ataxia and systemic immunological disorders

### Anti-GAD ataxia

Non-paraneoplastic autoimmune cerebellar ataxia was first defined some 20 years ago with the identification of antibodies, in both serum and CSF, directed against the enzyme glutamic acid decarboxylase (GAD) [1]. Since the earliest reports, a strong association between anti-GAD and systemic autoimmune conditions has been confirmed, including type 1 diabetes, pernicious anaemia, vitiligo and thyroiditis [2]. Yet, the cerebellum should in itself be considered a primary target organ for autoimmunity, as evidenced by the high proportion of HLA type DQ2 and the demonstration of cerebellar antibodies in some 60% of patients with otherwise idiopathic ataxia [3]. The cerebellum therefore, alongside the hippocampus, appears particularly vulnerable to immune-mediated pathology—mechanistic evidence is strongest for the GAD65 isoform, probably via (reversible) interference in GABA exocytosis (reviewed in [4•]) but also resulting in (irreversible) Purkinje cell loss [5]. Anti-GAD disease may present clinically with either sub-acute or chronic ataxia, potentially accompanied by features of overlapping neurological conditions also attributed to anti-GAD, including stiff-person syndrome [6]. Therapeutic approaches may therefore simultaneously need to target symptomatic relief of muscle rigidity with GABAergic agonists such as diazepam [7]. A meaningful impact on ataxic symptoms is less reliably achieved, although baclofen was reportedly of benefit for periodic alternating nystagmus [8]. Outside the context of anti-GAD disease, aminopyridines may relieve downbeat nystagmus [9] while acetyl-DL-leucine shows promise among unselected ataxia cases [10] but awaits placebo-controlled confirmation [11].

With the expectation that most patients with anti-GAD cerebellar ataxia will accumulate significant disability [12], consideration is often given to immunosuppression despite the lack of evidence from placebo-controlled trials [13]. Overall, the reported results of treatment are mixed, with the largest case series reporting ‘robust’ responses in a higher proportion of 55 non-paraneoplastic cerebellar ataxia patients compared with 63 paraneoplastic cases [14••]. Over half of the non-paraneoplastic cases in this series responded to immunotherapy, typically including long-term maintenance. Long-term follow-up data has also been reported for a further 25 ataxia patients with GAD65 antibodies in whom 35% experienced an improvement of at least 1 point on the modified Rankin Scale [15•]. Sub-acute presentations carried a more favourable prognosis than chronic ataxia. Varying induction regimes combining steroids, intravenous immunoglobulins (IVIg) and plasma exchange (PEx) have been utilised, typically relying on clinical status to assess response, although neurophysiological outcome markers may also inform [16]. While decreases in serum, anti-GAD levels have often tracked effective therapy, for example with rituximab [17], intrathecal levels may be more reflective of treatment response [18]. Further uncertainty surrounds cases of cerebellar ataxia with low (< 100 U/ml) serum titres of anti-GAD, in whom responses to immunotherapy are sparsely described [19, 20]. Antibodies against the GAD67 epitope may potentially also be pathogenic [21]. Evidence-based recommendations for maintenance therapy are not currently possible but previous choices have included azathioprine and mycophenolate (discussed in [22]).

### Gluten ataxia

Anti-GAD antibodies have also been detected in 40% of a well-defined cerebellar ataxia cohort curated in Sheffield, UK [23]*.* These patients, however, had an existing diagnosis of *gluten ataxia* suggesting therefore an overlapping spectrum of cerebellar autoimmunity. This neurological syndrome is infrequently the presenting clinical feature of coeliac disease, antibodies to gliadin and/or transglutaminase are accordingly detectable in between 10 and 50% of sporadic ataxia patients, depending on the background population [24]. Coeliac disease is also held responsible for cases of peripheral neuropathy [25] and other diverse neurological manifestations, yet, not all gluten ataxia patients have biopsy evidence of enteropathy and few have gastrointestinal symptoms; conversely 37% of newly diagnosed coeliac patients exhibit neurological signs in one report [26].

While randomised trial data concerning treatment of gluten ataxia is lacking, the broader necessity for systemic control of coeliac disease applies equally to this subgroup [27]. Strict adherence to a gluten-free diet is therefore recommended. A single pragmatically designed study investigated the impact on gluten ataxia on 26 patients who adhered to the diet compared at baseline to 14 patients who declined the intervention [28]. Diet-compliant patients, regardless of whether enteropathy was initially detectable, showed significant improvement across a range of ataxia clinical measures. Further mechanistic evidence in support of gluten avoidance is provided by a recent MR spectroscopy study in which a normalisation of NAA/Cr ratio in the cerebellar vermis resulted from successful diet adherence (as indexed by elimination of detectable anti-gliadin antibodies); the effect was diminished by partial adherence [29]. Symptomatic improvement is typically delayed by approximately 1 year and ataxia may only stabilise in advanced cases with established cerebellar atrophy. Whether incremental benefit is likely with simultaneous immunosuppression remains to be determined; isolated reports describe clinical improvement with IVIg [30], even among cases refractory to gluten-free diet [31].

## Ataxia and systemic endocrine/metabolic disorders

### Alcohol-induced cerebellar disease

Chronic excessive alcohol ingestion is a common cause of cerebellar degeneration. Clinically, patients present with ataxia predominantly affecting legs and gait, and typically occurs in association with features of malnutrition. Some patients may present acutely with Wernicke encephalopathy (WE), which has the added features of delirium, cognitive impairment and oculomotor deficits. The association of WE and thiamine (vitamin B_1_) deficiency is well-recognised; however, it remains unclear to what extent chronic alcohol-induced cerebellar damage is caused by the effects of thiamine deficiency or directly attributable to the toxic effects of alcohol on the brain [32]. Abstinence from alcohol and thiamine supplementation remains the mainstay of treatment.

Abstinence from alcohol was also shown to result in clinically relevant improvement in chronic alcoholic cerebellar degeneration, as assessed by measures of postural instability over 18 months [33]. A further study (over 2 years) confirmed that abstinence can improve postural stability and may be linked to changes in fourth ventricular size [34]. Perhaps not surprisingly, long-term abstinence (> 18 months) is associated with better recovery of gait and balance problems above and beyond short-term abstinence (6–15 weeks) [35].

Regarding thiamine replacement, its role in WE is established. However, a recent Cochrane review only found two randomised trials and no firm conclusion regarding the dose, route of administration and minimum duration for treatment or prophylaxis against WE could be made [36••]. In practice, due to unreliable absorption of thiamine in malnourished patients, it is recommended that thiamine is administered intravenously in acute WE, although only class IV evidence exists to back up this guidance [37]. Whether thiamine replacement is of benefit in chronic alcoholic ataxia is unknown, but typically, patients are prescribed with vitamin supplementation. The fact that WE can occur in the setting of poor nutrition unrelated to alcoholism suggests that thiamine replacement is likely to be of benefit in patients who have become abstinent, although evidence is lacking. There is no evidence that thiamine replacement is effective in improving ataxia in those with established chronic alcoholic cerebellar degeneration.

### Thyroid disease and ataxia

Ataxia has been reported in hypothyroidism [38]. However, whether a lack of thyroid hormone genuinely causes cerebellar dysfunction is not clear. Hypothyroidism is common and the rarity of ataxia in the condition may suggest a lack of causality and possibly coincidence of separate diseases [39]. Gait problems in hypothyroidism may often be attributed to the typical changes in muscle contractility that occurs. Treatment with thyroxine is routine and typically improves systemic features of the disease.

Hashimoto’s encephalopathy (HE) is a neurological condition associated with elevated anti-thyroid antibodies (anti-thyroglobulin or anti-thyroid peroxidase (TPO)), often occurring in euthyroid individuals [40]. Since anti-TPO antibodies can occur in the healthy general population, the diagnosis may be challenging. Antibodies against NH2-terminal of α-enolase (NAE) have been reported as a useful serum biomarker of HE [41]. Reports have suggested that HE may rarely present as an isolated ataxic syndrome. A case series of 13 adult patients (of whom eight had anti-NAE antibodies) with a predominantly ataxic form of HE reported that truncal ataxia is common and nystagmus is uncommon, and eight out of 13 had a syndrome mimicking spinocerebellar degeneration with insidious onset [42]. One of the cardinal features of HE is its responsiveness to immunotherapy (hence, the alternative nomenclature of steroid-responsive encephalopathy associated with autoimmune thyroiditis [SREAT]). A recent study of 13 patients with anti-NAE HE reported full or good recovery in 11 patients treated with methylprednisolone with or without prednisolone tail (one patient also received plasma exchange) [43]. In the case series of ataxic HE mentioned above, all patients responded to steroid treatment and/or IVIg [42]. Response appeared better in those with anti-NAE antibodies although the study size was small. Despite the usually good response to steroid therapy, relapse after steroid withdrawal may occur and optimal maintenance therapy is not clear. There is no trial evidence but reports have described use of azathioprine, mycophenolate mofetil, methotrexate, cyclophosphamide, rituximab, maintenance IVIg and plasma exchange [44–46].

## Paraneoplastic ataxic manifestations

Paraneoplastic syndromes within the central nervous system (CNS) refer to non-metastatic involvement of the brain and/or spinal cord, most likely occurring as a result of an immune reaction to cancer cells generating antibodies which cross-react with CNS antigens [47]. They occur rarely, but of the described CNS paraneoplastic syndromes, paraneoplastic cerebellar degeneration (PCD) is the commonest.

### Paraneoplastic cerebellar degeneration

Fifty to 60% of patients with paraneoplastic cerebellar degeneration (PCD) have identifiable antibodies in serum and/or CSF, of which anti-Yo (alternatively referred to as anti-Purkinje cell cytoplasmic antibody-1; PCA-1) is the commonest, representing approximately 50% of antibody-positive PCD [48]. PCD typically presents with sub-acute onset of ataxia which, often rapidly, progresses to pan-cerebellar failure. Often, cerebellar features plateau after a period of time. PCD may occur as an isolated cerebellar syndrome usually associated with breast or ovarian cancer and anti-Yo antibodies, or with Hodgkin’s lymphoma and Tr/DNER antibodies [49]. Alternatively, PCD presents in association with other neurological features such as encephalomyelitis (small cell lung cancer and anti-Hu antibodies) [50] or eye movement abnormalities (typically, opsoclonus with breast or ovarian cancer and anti-Ri antibodies) [51]. The repertoire of antibodies associated with PCD is increasing, now including antibodies against the Purkinje cell protein carbonic anhydrase-related protein VIII (CARP) [52] and protein kinase Cγ (PKCγ) [53]. It seems likely that further antibodies will be discovered identifying a cause in antibody-negative PCD cases.

In general, PCD responds poorly to treatment. The rarity of the condition, coupled to the multiple antibodies that may cause the phenotype, has meant that randomised controlled trials of PCD therapies are lacking. In addition, the rather rapid disease onset followed by plateau renders determination of treatment efficacy challenging.

Treatment or removal of the underlying tumour, if possible, is important, although even if successful, often, the symptoms of PCD do not improve and the majority of patients with PCD will remain severely disabled [50, 54]. Prognosis is poor and median survival in a study of 34 anti-Yo PCD cases was 22 months [54]. Studies have suggested that prognosis may be slightly better in PCD associated with antibodies other than anti-Yo [55]. Indeed, improvement in PCD associated with anti-Tr antibodies and Hodgkin’s lymphoma has been reported following treatment of the cancer [56].

Studies have assessed the utility of additional immunotherapy in PCD. The outcomes are generally disappointing although some small case series have reported benefit (class IV evidence). Immunotherapies such as corticosteroids, IVIg and PEx have been most studied. PEx has been reported to cause improvement in some cases, although the majority of reported cases do not appear to improve [57]. Similarly, IVIg has been reported to be effective in single cases, although a study of 22 patients with anti-neuronal antibody-associated paraneoplastic neurological syndromes (including 4 with PCD) did not reveal any effect on disability using IVIg (median 5.8 cycles) [58–62]. The most commonly reported alternative immunosuppressant is cyclophosphamide, but, again, evidence is limited only to single cases [63, 64]. Aggressive combination immunosuppressive regimes have been reported [65]. A follow-up study of 17 patients with anti-Yo and anti-Hu paraneoplastic encephalomyelitis, sensory neuropathy or PCD receiving cycles of IVIg, cyclophosphamide and methyl prednisolone reported clinical stabilisation (evaluated by the modified Rankin scale) in those patients who were not severely disabled at time of treatment onset, but no effect in severely disabled PCD patients [66]. It remains unclear whether early administration of immunosuppression in PCD improves prognosis.

Newer therapies including tacrolimus and rituximab have been studied in case reports or small case series in PCD. Rituximab has been reported to induce partial remission in individual cases [67–69]. Tacrolimus, a potent T cell inhibitor, has been studied in a series of patients with paraneoplastic neurological disease (including PCD) [70]. Although, the trial was not designed to quantify neurological outcome, there were anecdotal reports in some patients of improvement in function. More studies are required to determine the influence of immunosuppressive regimes on PCD.

## Conclusion

There is some evidence concerning treatment regimens for cerebellar ataxias occurring in the context of systemic disease. Cerebellar ataxia occurring in the context of a systemic immunological disorder is often amenable to a range of immunomodulatory therapies, although the rarity of these disorders and the phenotypic variability makes defining optimal treatment regimens challenging. The commoner ataxic manifestations of metabolic disorders, notably alcohol-induced cerebellar injury, have been the subject of several studies, although there is a paucity of evidence concerning optimal treatments for chronic alcoholic cerebellar degeneration. Despite their rarity, cerebellar ataxias occurring in the context of systemic disease cause significant morbidity and better therapies are required to improve outcomes associated with these conditions.

## References

[1] Honnorat J, Trouillas P, Thivolet C, Aguera M, Belin MF (1995). Autoantibodies to glutamate decarboxylase in a patient with cerebellar cortical atrophy, peripheral neuropathy, and slow eye movements. Arch Neurol.

[2] Barova H, Perusicova J, Hill M, Sterzl I, Vondra K, Masek Z (2004). Anti-GAD-positive patients with type 1 diabetes mellitus have higher prevalence of autoimmune thyroiditis than anti-GAD-negative patients with type 1 and type 2 diabetes mellitus. Physiol Res.

[3] Hadjivassiliou M, Boscolo S, Tongiorgi E, Grunewald RA, Sharrack B, Sanders DS (2008). Cerebellar ataxia as a possible organ-specific autoimmune disease. Mov Disord.

[4] Mitoma H, Adhikari K, Aeschlimann D, Chattopadhyay P, Hadjivassiliou M, Hampe CS (2016). Consensus paper: neuroimmune mechanisms of cerebellar ataxias. Cerebellum.

[5] Ishida K, Mitoma H, Wada Y, Oka T, Shibahara J, Saito Y (2007). Selective loss of Purkinje cells in a patient with anti-glutamic acid decarboxylase antibody-associated cerebellar ataxia. J Neurol Neurosurg Psychiatry.

[6] Baizabal-Carvallo JF, Jankovic J (2015). Stiff-person syndrome: insights into a complex autoimmune disorder. J Neurol Neurosurg Psychiatry.

[7] McKeon A, Robinson MT, McEvoy KM, Matsumoto JY, Lennon VA, Ahlskog JE (2012). Stiff-man syndrome and variants: clinical course, treatments, and outcomes. Arch Neurol.

[8] Tilikete C, Vighetto A, Trouillas P, Honnorat J (2005). Anti-GAD antibodies and periodic alternating nystagmus. Arch Neurol.

[9] Glasauer S, Strupp M, Kalla R, Buttner U, Brandt T (2005). Effect of 4-aminopyridine on upbeat and downbeat nystagmus elucidates the mechanism of downbeat nystagmus. Ann N Y Acad Sci.

[10] Strupp M, Teufel J, Habs M, Feuerecker R, Muth C, van de Warrenburg BP (2013). Effects of acetyl-DL-leucine in patients with cerebellar ataxia: a case series. J Neurol.

[11] Feil K, Adrion C, Teufel J, Bosch S, Claassen J, Giordano I (2017). Effects of acetyl-DL-leucine on cerebellar ataxia (ALCAT trial): study protocol for a multicenter, multinational, randomized, double-blind, placebo-controlled, crossover phase III trial. BMC Neurol.

[12] Honnorat J, Saiz A, Giometto B, Vincent A, Brieva L, de Andres C (2001). Cerebellar ataxia with anti-glutamic acid decarboxylase antibodies: study of 14 patients. Arch Neurol.

[13] Mitoma H, Hadjivassiliou M, Honnorat J (2015). Guidelines for treatment of immune-mediated cerebellar ataxias. Cerebellum Ataxias.

[14] Jones AL, Flanagan EP, Pittock SJ, Mandrekar JN, Eggers SD, Ahlskog JE (2015). Responses to and outcomes of treatment of autoimmune cerebellar ataxia in adults. JAMA Neurol..

[15] Arino H, Gresa-Arribas N, Blanco Y, Martinez-Hernandez E, Sabater L, Petit-Pedrol M (2014). Cerebellar ataxia and glutamic acid decarboxylase antibodies: immunologic profile and long-term effect of immunotherapy. JAMA Neurol..

[16] Simon NG, Vucic S, Joffe R, Kiernan MC (2014). Cortical dysfunction in cerebellar ataxia with antibodies to glutamic acid decarboxylase. J Neurol.

[17] Planche V, Marques A, Ulla M, Ruivard M, Durif F (2014). Intravenous immunoglobulin and rituximab for cerebellar ataxia with glutamic acid decarboxylase autoantibodies. Cerebellum.

[18] McFarland NR, Login IS, Vernon S, Burns TM (2006). Improvement with corticosteroids and azathioprine in GAD65-associated cerebellar ataxia. Neurology.

[19] Virgilio R, Corti S, Agazzi P, Santoro D, Lanfranconi S, Candelise L (2009). Effect of steroid treatment in cerebellar ataxia associated with anti-glutamic acid decarboxylase antibodies. J Neurol Neurosurg Psychiatry.

[20] Nanri K, Niwa H, Mitoma H, Takei A, Ikeda J, Harada T (2013). Low-titer anti-GAD-antibody-positive cerebellar ataxia. Cerebellum.

[21] Guasp M, Sola-Valls N, Martinez-Hernandez E, Gil MP, Gonzalez C, Brieva L (2016). Cerebellar ataxia and autoantibodies restricted to glutamic acid decarboxylase 67 (GAD67). J Neuroimmunol.

[22] Baizabal-Carvallo JF, Alonso-Juarez M. Cerebellar disease associated with anti-glutamic acid decarboxylase antibodies: review. J Neural Transm (Vienna). 2017;124(10):1171–1182. 10.1007/s00702-017-1754-3. 10.1007/s00702-017-1754-3 28689294

[23] Hadjivassiliou M, Aeschlimann D, Grunewald RA, Sanders DS, Sharrack B, Woodroofe N (2011). GAD antibody-associated neurological illness and its relationship to gluten sensitivity. Acta Neurol Scand.

[24] Hadjivassiliou M, Sanders DS, Grunewald RA, Woodroofe N, Boscolo S, Aeschlimann D (2010). Gluten sensitivity: from gut to brain. Lancet Neurol.

[25] Thawani SP, Brannagan TH, Lebwohl B, Green PH, Ludvigsson JF (2015). Risk of neuropathy among 28,232 patients with biopsy-verified celiac disease. JAMA Neurol..

[26] Currie S, Hoggard N, Sanders D, Wilkinson I, Griffiths P, Hadjivassiliou M (2014). Coeliac disease and neurological dysfunction: a case-control study. Lancet.

[27] Ludvigsson JF, Bai JC, Biagi F, Card TR, Ciacci C, Ciclitira PJ (2014). Diagnosis and management of adult coeliac disease: guidelines from the British Society of Gastroenterology. Gut.

[28] Hadjivassiliou M, Davies-Jones GA, Sanders DS, Grunewald RA (2003). Dietary treatment of gluten ataxia. J Neurol Neurosurg Psychiatry.

[29] Hadjivassiliou M, Grunewald RA, Sanders DS, Shanmugarajah P, Hoggard N. Effect of gluten-free diet on cerebellar MR spectroscopy in gluten ataxia. Neurology. 2017;89(7):705–709. 10.1212/WNL.0000000000004237. 10.1212/WNL.0000000000004237 28724585

[30] Nanri K, Okuma M, Sato S, Yoneda M, Taguchi T, Mitoma H (2016). Prevalence of autoantibodies and the efficacy of immunotherapy for autoimmune cerebellar ataxia. Intern Med.

[31] Souayah N, Chin RL, Brannagan TH, Latov N, Green PH, Kokoszka A (2008). Effect of intravenous immunoglobulin on cerebellar ataxia and neuropathic pain associated with celiac disease. Eur J Neurol.

[32] Victor M, Adams RD (1961). On the etiology of the alcoholic neurologic diseases with special reference to the role of nutrition. Am J Clin Nutr.

[33] Diener HC, Dichgans J, Bacher M, Guschlbauer B (1984). Improvement of ataxia in alcoholic cerebellar atrophy through alcohol abstinence. J Neurol.

[34] Rosenbloom MJ, Rohlfing T, O'Reilly AW, Sassoon SA, Pfefferbaum A, Sullivan EV (2007). Improvement in memory and static balance with abstinence in alcoholic men and women: selective relations with change in brain structure. Psychiatry Res.

[35] Smith S, Fein G (2011). Persistent but less severe ataxia in long-term versus short-term abstinent alcoholic men and women: a cross-sectional analysis. Alcohol Clin Exp Res.

[36] •• Day E, Bentham PW, Callaghan R, Kuruvilla T, George S. Thiamine for prevention and treatment of Wernicke-Korsakoff Syndrome in people who abuse alcohol. Cochrane Database Syst Rev. 2013 Jul 1;(7):CD004033. 10.1002/14651858.CD004033.pub3. Cochrane database review on studies of thiamine in WE. 10.1002/14651858.CD004033.pub3 PMC7163251 23818100

[37] Galvin R, Brathen G, Ivashynka A, Hillbom M, Tanasescu R, Leone MA (2010). EFNS guidelines for diagnosis, therapy and prevention of Wernicke encephalopathy. Eur J Neurol.

[38] Sandyk R (1982). Cerebellar dysfunction in hypothyroidism. S Afr Med J.

[39] Quinn N, Barnard RO, Kelly RE (1992). Cerebellar syndrome in myxoedema revisited: a published case with carcinomatosis and multiple system atrophy at necropsy. J Neurol Neurosurg Psychiatry.

[40] Shaw PJ, Walls TJ, Newman PK, Cleland PG, Cartlidge NE (1991). Hashimoto's encephalopathy: a steroid-responsive disorder associated with high anti-thyroid antibody titers—report of 5 cases. Neurology.

[41] Yoneda M, Fujii A, Ito A, Yokoyama H, Nakagawa H, Kuriyama M (2007). High prevalence of serum autoantibodies against the amino terminal of alpha-enolase in Hashimoto’s encephalopathy. J Neuroimmunol.

[42] Matsunaga A, Ikawa M, Fujii A, Nakamoto Y, Kuriyama M, Yoneda M (2013). Hashimoto’s encephalopathy as a treatable adult-onset cerebellar ataxia mimicking spinocerebellar degeneration. Eur Neurol.

[43] Kishitani T, Matsunaga A, Ikawa M, Hayashi K, Yamamura O, Hamano T (2017). Limbic encephalitis associated with anti-NH2-terminal of alpha-enolase antibodies: a clinical subtype of Hashimoto encephalopathy. Medicine (Baltimore).

[44] Olmez I, Moses H, Sriram S, Kirshner H, Lagrange AH, Pawate S (2013). Diagnostic and therapeutic aspects of Hashimoto’s encephalopathy. J Neurol Sci.

[45] Marshall GA, Doyle JJ (2006). Long-term treatment of Hashimoto’s encephalopathy. J Neuropsychiatry Clin Neurosci.

[46] Laurent C, Capron J, Quillerou B, Thomas G, Alamowitch S, Fain O (2016). Steroid-responsive encephalopathy associated with autoimmune thyroiditis (SREAT): characteristics, treatment and outcome in 251 cases from the literature. Autoimmun Rev.

[47] Hoftberger R, Rosenfeld MR, Dalmau J (2015). Update on neurological paraneoplastic syndromes. Curr Opin Oncol.

[48] Venkatraman A, Opal P (2016). Paraneoplastic cerebellar degeneration with anti-Yo antibodies—a review. Ann Clin Transl Neurol.

[49] Greene M, Lai Y, Baella N, Dalmau J, Lancaster E (2014). Antibodies to Delta/notch-like epidermal growth factor-related receptor in patients with anti-Tr, paraneoplastic cerebellar degeneration, and Hodgkin lymphoma. JAMA Neurol.

[50] Shams'ili S, Grefkens J, de Leeuw B, van den Bent M, Hooijkaas H, van der Holt B (2003). Paraneoplastic cerebellar degeneration associated with antineuronal antibodies: analysis of 50 patients. Brain.

[51] Pittock SJ, Lucchinetti CF, Lennon VA (2003). Anti-neuronal nuclear autoantibody type 2: paraneoplastic accompaniments. Ann Neurol.

[52] Bataller L, Sabater L, Saiz A, Serra C, Claramonte B, Graus F (2004). Carbonic anhydrase-related protein VIII: autoantigen in paraneoplastic cerebellar degeneration. Ann Neurol.

[53] Sabater L, Bataller L, Carpentier AF, Aguirre-Cruz ML, Saiz A, Benyahia B (2006). Protein kinase Cgamma autoimmunity in paraneoplastic cerebellar degeneration and non-small-cell lung cancer. J Neurol Neurosurg Psychiatry.

[54] Rojas I, Graus F, Keime-Guibert F, Rene R, Delattre JY, Ramon JM (2000). Long-term clinical outcome of paraneoplastic cerebellar degeneration and anti-Yo antibodies. Neurology.

[55] Giometto B, Grisold W, Vitaliani R, Graus F, Honnorat J, Bertolini G (2010). Paraneoplastic neurologic syndrome in the PNS Euronetwork database: a European study from 20 centers. Arch Neurol.

[56] Briani C, Vitaliani R, Grisold W, Honnorat J, Graus F, Antoine JC (2011). Spectrum of paraneoplastic disease associated with lymphoma. Neurology.

[57] Peterson K, Rosenblum MK, Kotanides H, Posner JB (1992). Paraneoplastic cerebellar degeneration. I. A clinical analysis of 55 anti-Yo antibody-positive patients. Neurology.

[58] Moll JW, Henzen-Logmans SC, Van der Meche FG, Vecht CH (1993). Early diagnosis and intravenous immune globulin therapy in paraneoplastic cerebellar degeneration. J Neurol Neurosurg Psychiatry.

[59] Counsell CE, McLeod M, Grant R (1994). Reversal of subacute paraneoplastic cerebellar syndrome with intravenous immunoglobulin. Neurology.

[60] Uchuya M, Graus F, Vega F, Rene R, Delattre JY (1996). Intravenous immunoglobulin treatment in paraneoplastic neurological syndromes with antineuronal autoantibodies. J Neurol Neurosurg Psychiatry.

[61] Phuphanich S, Brock C (2007). Neurologic improvement after high-dose intravenous immunoglobulin therapy in patients with paraneoplastic cerebellar degeneration associated with anti-Purkinje cell antibody. J Neuro-Oncol.

[62] Schessl J, Schuberth M, Reilich P, Schneiderat P, Strigl-Pill N, Walter MC (2011). Long-term efficiency of intravenously administered immunoglobulin in anti-Yo syndrome with paraneoplastic cerebellar degeneration. J Neurol.

[63] Thone J, Hohaus A, Lamprecht S, Bickel A, Erbguth F (2008). Effective immunosuppressant therapy with cyclophosphamide and corticosteroids in paraneoplastic cerebellar degeneration. J Neurol Sci.

[64] Stark E, Wurster U, Patzold U, Sailer M, Haas J (1995). Immunological and clinical response to immunosuppressive treatment in paraneoplastic cerebellar degeneration. Arch Neurol.

[65] Mowzoon N, Bradley WG (2000). Successful immunosuppressant therapy of severe progressive cerebellar degeneration and sensory neuropathy: a case report. J Neurol Sci.

[66] Keime-Guibert F, Graus F, Fleury A, Rene R, Honnorat J, Broet P, et al. Treatment of paraneoplastic neurological syndromes with antineuronal antibodies (anti-Hu, anti-Yo) with a combination of immunoglobulins, cyclophosphamide, and methylprednisolone. J Neurol Neurosurg Psychiatry. 2000;68(4):479–82. 10.1136/jnnp.68.4.479 PMC1736897 10727484

[67] Esposito M, Penza P, Orefice G, Pagano A, Parente E, Abbadessa A (2008). Successful treatment of paraneoplastic cerebellar degeneration with rituximab. J Neuro-Oncol.

[68] Shams'ili S, de Beukelaar J, Gratama JW, Hooijkaas H, van den Bent M, van 't Veer M (2006). An uncontrolled trial of rituximab for antibody associated paraneoplastic neurological syndromes. J Neurol.

[69] Yeo KK, Walter AW, Miller RE, Dalmau J (2012). Rituximab as potential therapy for paraneoplastic cerebellar degeneration in pediatric Hodgkin disease. Pediatr Blood Cancer.

[70] Orange D, Frank M, Tian S, Dousmanis A, Marmur R, Buckley N (2012). Cellular immune suppression in paraneoplastic neurologic syndromes targeting intracellular antigens. Arch Neurol.

